# Dietary weight loss‐induced changes in RBP4, FFA, and ACE predict weight regain in people with overweight and obesity

**DOI:** 10.14814/phy2.13450

**Published:** 2017-11-09

**Authors:** Roel G. Vink, Nadia J. Roumans, Edwin C. Mariman, Marleen A. van Baak

**Affiliations:** ^1^ Department of Human Biology NUTRIM School of Nutrition and Translational Research in Metabolism Maastricht University Medical Centre^+^ Maastricht the Netherlands

**Keywords:** Adipokines, endocrinology, obesity, weight loss, weight regain

## Abstract

Adipokines and other biomarkers were previously identified with roles in energy expenditure, appetite, satiety, and adiposity. Therefore, we investigated whether dietary weight loss‐induced changes in adipokines and other biomarkers known to play a role in weight regulation or energy expenditure could predict weight regain in people with overweight and obesity. In this randomized controlled trial 26 males and 30 females (BMI: 28–35 kg/m^2^) followed either a low‐calorie diet (LCD; 1250 kcal/day) for 12 weeks or a very‐low‐calorie diet (VLCD; 500 kcal/day) for 5 weeks followed by a weight stable period of 4 weeks (dietary intervention (DI) period) and a 9‐month follow‐up period. Blood samples were taken before and after each period to measure FFA, TAG, total cholesterol, insulin, glucose, angiotensin‐converting enzyme (ACE) activity, IL‐6, RBP4, apelin, leptin, adiponectin, vaspin, and nesfatin‐1 concentrations. Weight loss was similar between groups (LCD: −8.2 ± 0.5 kg; VLCD: −8.9 ± 0.4 kg, *P *=* *0.30). Only changes in ACE activity, FFA and RBP4 concentrations during DI were correlated with weight regain in the whole group (*r* = −0.299, *P *=* *0.030, *r* = −0.274, *P = *0.047, and *r* = 0.357, *P *=* *0.008, respectively). Together they explained 28% (*r* = 0.532) of weight regain variation. Dietary weight loss‐induced changes in ACE activity, FFA and RBP4 independently contribute to weight regain prediction.

## Introduction

Worldwide there were over 2.1 billion people with overweight and obesity in 2013 and in 2010 was estimated to cause 3.4 million deaths (Ng et al. 2014). A dietary intervention achieving 5–10% weight loss reduces disease risk and produces positive health outcomes in people with overweight and obesity (Van Gaal et al. 1997; Weinstock et al. 1998).

Individuals with obesity that follow an energy‐restricted dietary intervention commonly achieve significant weight loss. However, long‐term weight maintenance results are often poor (Sarlio‐Lahteenkorva et al. 2000; Weiss et al. 2007). For instance, it was recently shown that participants regained ~70% of lost weight within 2 years after the dietary intervention (Purcell et al. 2014). Besides the important behavioral and psychological aspects of obesity there is now substantial evidence that biological and metabolic mechanisms contribute to or can even drive weight regain after weight loss (Maclean et al. 2011; Mariman 2012; Roumans et al. 2015). The adipose tissue (AT) produces hormones and metabolic substrates that can affect energy expenditure, appetite, satiety, and fat distribution (Kloting et al. 2011; Bluher 2014), which could offer opportunities for the development of anti‐obesity drugs (Bluher and Mantzoros 2015). However, the limited human data on these anti‐obesity candidates remains one of the major road blocks in drug discovery (Bluher 2014). Furthermore, the identification of molecules that predict weight regain and/or improvement of the metabolic profile could help to identify individuals with increased risk of weight regain and may represent interesting targets for prevention of weight regain. Furthermore, we have previously shown that the rate of weight loss influences the diet‐induced loss of fat‐free mass (Vink et al. 2016), yet it is unknown how the rate of weight loss affects these molecules.

Therefore, we selected several adipokines and other biomarkers that were previously shown to be correlated with weight regain or had the potential to influence weight regain due to their functions in energy expenditure, food intake and/or body weight regulation (FFA, TAG, total cholesterol, insulin, glucose, HOMA‐IR, angiotensin‐converting enzyme (ACE) activity, interleukin‐6 (IL‐6), retinol‐binding protein 4 (RBP4), apelin, leptin, adiponectin, vaspin, nesfatin‐1, and adipocyte size). The objective of this study was to investigate whether diet‐induced changes in these adipokines and other biomarkers could predict weight regain following weight loss in people with overweight and obesity and whether the rate of weight loss modifies this prediction.

## Methods

### Subjects

Sixty‐one individuals with overweight and obesity (BMI 28–35 kg/m^2^) were recruited by advertisement via local media. Exclusion criteria, as described previously (Vink et al. 2016), were smoking, cardiovascular disease, type 2 diabetes mellitus, liver or kidney disease, use of medication that influences body weight regulation, pregnancy, marked alcohol consumption (>21 alcoholic units per week for men and >14 alcoholic units per week for women), elevated fasting glucose (>6.1 mmol/L), total cholesterol (>7.0 mmol/L) or triacylglycerol (>3.0 mmol/L) concentrations, or blood pressure (>160/100 mmHg). Furthermore, participants had to be weight stable (weight change <3.0 kg) for 2 months prior to the start of the study. All participants gave their written informed consent before participation in the study. The study was performed according to the declaration of Helsinki and was approved by the Medical Ethics Committee of Maastricht University Medical Centre.

### Experimental protocol

**Figure 1:**
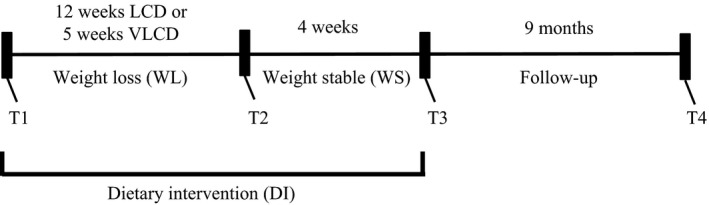
Study overview. Fasting serum and plasma blood samples were taken at study start (T1), end of the weight loss (WL) period (T2), end of the weight stable (WS) period (T3), and end of the follow‐up period (T4). The WL period and WS period together was named as the dietary intervention (DI) period.

The experimental protocol has been described previously (Vink et al. 2016). Briefly, the participants followed a dietary intervention program that was divided in three periods (Fig. 1). First, participants were randomized to a 5‐week very‐low‐calorie diet (VLCD) or a 12‐week low‐calorie diet (LCD) (weight loss (WL) period, T2–T1) and subsequently underwent a 4‐week weight stable (WS, T3–T2) period. The third period consisted of a 9‐month follow‐up period (T4–T3). The WL and WS periods taken together were named the dietary intervention (DI, T3–T1) period. The WS period was included to investigate the effects of approximately 10% weight loss, without the presence of a negative energy balance. Participants in the LCD group underwent a 12‐week diet providing 1250 kcal/day designed by a dietician. In the VLCD group participants underwent a 5‐week diet in which three meals per day were replaced by meal replacements, providing 500 kcal/day. Both groups subsequently underwent a 4‐week WS period with a diet based on the energy requirements of the participants. The study dietician provided dietary advice to both groups to assist in weight loss during the WL period (five meetings) and in remaining weight stable throughout the WS period (four meetings). Body weight and blood pressure were measured monthly at the Maastricht University Medical Centre for 9 months during follow‐up. However, dietary advice was no longer given to mimic non‐restricted free‐living conditions.

At T1, T2, T3, and T4 blood samples were drawn, abdominal subcutaneous AT biopsies were collected, body composition was determined and body weight, height, blood pressure and waist and hip circumference were measured after an overnight fast of at least 10 h. At T1, T3, and T4 a standard OGTT was performed to determine 2‐h glucose values. Subjects were enrolled between February 2012 – January 2015. Because of the obvious differences between the dietary interventions in this study, the researchers, study participants and dietician were not blinded to the intervention. This trial is registered with www.clinicaltrials.gov: as NCT01559415.

### Anthropometric measurements

Body weight, height, waist and hip circumference and blood pressure were measured as described previously (Vink et al. 2016). Body composition was determined with the Bod Pod device (Cosmed, Italy, Rome) as described previously (Vink et al. 2016).

### Adipose tissue biopsy

Abdominal subcutaneous AT needle biopsies (≈1 g) were collected 6–8 cm lateral from the umbilicus under local anesthesia (2% lidocaine) after an overnight fast of at least 10 h at T1, T2, T3, and T4. Biopsies were immediately rinsed with sterile saline and visible blood vessels were removed with sterile tweezers. A small sample of AT was fixed overnight in 4% paraformaldehyde and embedded in paraffin for analysis of adipocyte size.

### Adipocyte size

Histological sections (8 *μ*m) were cut from paraffin‐embedded tissue, mounted on microscope glass slides, and dried overnight in an incubator at 37°C. Sections were stained with hematoxylin and eosin. Digital images were captured with the use of a Leica DFC320 digital camera (Leica, Rijswijk, the Netherlands) at 20× magnification (Leica DM3000 microscope, Leica, Rijswijk, the Netherlands). Computerized morphometric analysis (Leica QWin V3, Cambridge, UK) of individual adipocytes was performed by measuring approximately 400 adipocytes per sample.

### Plasma analysis

Blood samples were collected into EDTA and serum tubes and were centrifuged for 15 min at 1000***g*** at 4°C and 21°C, respectively. Aliquots were immediately frozen in liquid nitrogen and subsequently stored at −80°C until analysis. Plasma glucose, FFA, TAG and total cholesterol were analyzed with standard enzymatic methods (ABX Pentra 400 autoanalyzer, Horiba ABX, Montpellier, France). Plasma glycerol was analyzed with an enzymatic assay (Enzytec Glycerol, Roche Biopharm, Basel, Switzerland) automated on a Cobas Fara spectrophotometric autoanalyzer. Plasma insulin, leptin, and adiponectin concentrations were analyzed with commercially available radioimmunoassay (RIA) kits (Human insulin specific RIA, human leptin RIA, human adiponectin RIA, Millipore Corporation, Billerica, MA). Plasma concentrations of IL‐6 (Meso Scale Discovery, Gaithersburg, MD), apelin‐12 (Phoenix pharmaceuticals Inc., Burlingame, CA), RBP4 (R&D systems, Minneapolis, MN) and Vaspin (Adipogen, San Diego, IL) were determined by ELISA. The apelin‐12 ELISA has 100% cross‐reactivity with human apelin‐12, apelin‐13, and apelin‐36. Serum ACE activity was measured with a standard enzymatic assay (Bühlmann, Basel, Switzerland) while serum nesfatin‐1 (Phoenix pharmaceuticals Inc., Burlingame, CA) concentrations were measured by ELISA.

### Calculations

Weight regain during follow up was calculated by dividing weight regain during follow‐up (T4–T3) by body weight at T3, multiplied by 100. Values of potential predictors of weight regain to be used in the correlation analysis (concentrations of FFA, TAG, total cholesterol, insulin, glucose, HOMA‐IR, ACE activity, IL‐6, RBP4, apelin, leptin, adiponectin, vaspin and nesfatin‐1, and the change in adipocyte size) were calculated by dividing change in concentration during the DI period (T3–T1) by the concentration at T1, multiplied by 100.

### Statistics

Data are presented as mean ± SEM. Comparisons of variables between time points within the same group were made with the paired‐sample *t*‐test. Between‐group comparisons were made with the independent‐samples *t*‐test. Correlations were calculated with Pearson (normally distributed) and Spearman's rank (non‐normally distributed) correlation coefficients. Variables were evaluated for residual normality and logarithmic transformations were performed when appropriate. Missing values were imputed by taking the value of the previous time point compared to the missing time point and adding the group‐based average change between these time points. Comparisons of the diet‐induced changes in adipokines and other biomarkers with time (four time points) by intervention group (two groups) (interaction: time × diet) were analyzed with a repeated measures analysis of variance (ANOVA). Post hoc analyses were performed with Bonferroni correction when a significant interaction of time × diet was observed, whereas presented *P*‐values represent Bonferroni corrected values. To assess whether multiple adipokines or other biomarkers could enhance weight regain prediction we used backward elimination in multiple linear regression. Statistical calculations were performed with SPSS for Macintosh, Version 22 (Chicago, IL). *P *<* *0.05 was considered statistically significant.

## Results

Four participants withdrew from the study during the DI period (three in VLCD group and one in LCD group) and blood samples were unavailable for one participant. Two participants started prescription medication that could influence body weight during follow‐up and were excluded from follow‐up analysis.

### Dietary weight loss‐induced changes in body weight, adipocyte size, adipokines, and other biomarkers

Participant characteristics at study start and diet‐induced changes in characteristics were published previously for all participants (Vink et al. 2016), whereas the results of all participants with available blood samples (*n* = 56) per diet group are shown in Table 1 and for the total group in Table 2. Weight loss during the WL period (T2–T1) was similar between groups (LCD: −8.2 ± 0.5 kg; VLCD: −8.9 ± 0.4 kg, *P *=* *0.30, Table 1) and body weight did not change significantly in either group in the subsequent 4 weeks of the weight stable period (T3–T2) (Table 1). Adipocyte size decreased during WL and significantly increased in the subsequent WS period and follow‐up period (Table 2). Weight regain (%) during follow‐up was similar between the LCD and VLCD group (5.1 ± 0.7% vs. 5.5 ± 0.9%, respectively, *P *=* *0.71).

**Table 1:** Characteristics of study participants in the LCD and VLCD groups

	Group	T1 (Study start)	T2 (End of WL)	T3 (End of WS)	T4 (End of follow‐up)	Diet × time (*P*‐value)
Sex (female/male)	LCD VLCD	15/14 15/12				
Age (year)	LCD VLCD	51.8 ± 1.9 50.8 ± 1.5				
Weight (kg)	LCD VLCD	92.4 ± 1.9 92.0 ± 1.8	84.2 ± 1.9 83.1 ± 1.6	84.0 ± 1.9 82.8 ± 1.7	88.4 ± 2.0 87.7 ± 2.0	0.670
BMI (kg/m^2^)	LCD VLCD	31.3 ± 0.5 30.8 ± 0.4	28.6 ± 0.5 27.9 ± 0.4	28.5 ± 0.5 27.8 ± 0.4	30.0 ± 0.5 29.1 ± 0.5	0.769
Waist circumference (cm)	LCD VLCD	102.6 ± 2.0 101.3 ± 1.5	95.3 ± 1.8 93.6 ± 1.4	94.3 ± 2.0 94.6 ± 1.3	98.3 ± 1.9 97.6 ± 1.6	0.266
Fat mass (%)	LCD VLCD	39.9 ± 1.8 39.7 ± 1.6	34.5 ± 2.1 35.1 ± 1.9	33.8 ± 2.1 33.9 ± 1.9	36.5 ± 2.0 36.3 ± 1.9	0.298
Fat‐free mass (kg)	LCD VLCD	55.4 ± 2.2 55.5 ± 2.3	54.9 ± 2.1 53.9 ± 2.2	55.2 ± 2.2 54.7 ± 2.3	56.0 ± 2.3 55.7 ± 2.3	0.034
Systolic blood pressure (mmHg)	LCD VLCD	126.8 ± 2.4 122.9 ± 2.8	123.7 ± 2.3 115.6 ± 2.5	124.1 ± 2.1 117.1 ± 2.8	127.4 ± 2.3 121.6 ± 3.0	0.474
Diastolic blood pressure (mmHg)	LCD VLCD	85.9 ± 1.9 84.4 ± 2.0	81.1 ± 1.5 78.0 ± 1.7	81.0 ± 1.5 78.2 ± 1.9	83.1 ± 1.8 82.9 ± 1.9	0.392
Glucose (mmol/L)	LCD VLCD	5.3 ± 0.1 5.2 ± 0.1	5.1 ± 0.1* 4.8 ± 0.1	5.1 ± 0.1* 5.0 ± 0.1	5.0 ± 0.1 4.9 ± 0.1	0.015
2‐h glucose (mmol/L)	LCD VLCD	5.9 ± 0.3 6.6 ± 0.4		5.0 ± 0.2† 6.8 ± 0.5	5.8 ± 0.4‡ 5.3 ± 0.2	0.000
Insulin (uU/mL)	LCD VLCD	17.1 ± 1.1 14.4 ± 1.2	12.4 ± 0.7 10.5 ± 1.1	13.4 ± 0.8 12.8 ± 1.4	12.8 ± 1.3 13.2 ± 1.9	0.195
FFA (*μ*mol/L)	LCD VLCD	496 ± 32 414 ± 30	559 ± 38.8** 763 ± 56	477 ± 45** 413 ± 35	531 ± 36 555 ± 38	0.000
TAG (*μ*mol/L)	LCD VLCD	1217 ± 98 1174 ± 90	947 ± 82 775 ± 55	1014 ± 82 1023 ± 65	977 ± 67 1013 ± 82	0.171
Total cholesterol (mmol/L)	LCD VLCD	6.0 ± 0.2 5.9 ± 0.2	5.8 ± 0.2 4.9 ± 0.1	5.8 ± 0.2* 5.7 ± 0.2	5.8 ± 0.1 6.0 ± 0.2	0.000
Adipocyte size (*μ*m)	LCD VLCD	68.2 ± 1.0 68.7 ± 1.0	61.3 ± 0.8 62.8 ± 1.2	65.9 ± 0.8 65.2 ± 1.2	67.0 ± 0.9 67.8 ± 1.0	0.342

Values are mean ± SEM. Post hoc results for significant diet × time interactions are shown with Bonferroni corrections. WL, weight loss; WS, weight stable; LCD, low‐calorie diet; VLCD, very‐low‐calorie diet.

*P <* *0.05, **P <* *0.01 difference in change between this time point and previous time point between dietary groups.

^†^ *P *<* *0.05 difference in change T3–T1 (DI period) between dietary groups, ^‡^ *P *<* *0.05 difference in change T4–T3 (follow‐up) between dietary groups.

**Table 2:** Participant characteristics and adipokines and other biomarkers in the whole group at T1, T2, T3, and T4

	T1 (Study start)	T2 (End of WL)	T3 (End of WS)	T4 (End of follow‐up)
Sex (male/female)	29/27			
Age (years)	51.3 ± 1.2			
Weight (kg)	92.2 ± 1.3	83.7 ± 1.2**	83.4 ± 1.3‡	88.1 ± 1.4**
BMI (kg/m^2^)	31.1 ± 0.3	28.2 ± 0.3**	28.1 ± 0.3‡	29.6 ± 0.4**
Waist circumference (cm)	102.0 ± 1.3	94.4 ± 1.1**	94.4 ± 1.2‡	98.0 ± 1.3**
Fat mass (%)	39.8 ± 1.2	34.8 ± 1.4**	33.8 ± 1.4**, ‡	36.4 ± 1.4**
Fat‐free mass (kg)	55.4 ± 1.6	54.4 ± 1.5**	55.0 ± 1.6‡	55.8 ± 1.6*
Systolic blood pressure (mmHg)	124.9 ± 1.8	119.8 ± 1.8**	120.7 ± 1.8‡	124.7 ± 1.9**
Diastolic blood pressure (mmHg)	85.2 ± 1.4	79.6 ± 1.1**	79.6 ± 1.2‡	83.0 ± 1.3**
Glucose (mmol/L)	5.2 ± 0.1	5.0 ± 0.1**	5.1 ± 0.1*, ‡	5.0 ± 0.1*
2‐h glucose (mmol/L)	6.3 ± 0.2		5.8 ± 0.3†	5.5 ± 0.2**
Insulin (*μ*U/mL)	15.8 ± 0.8	11.5 ± 0.6**	13.1 ± 0.8*, ‡	13.0 ± 1.1
FFA (*μ*mol/L)	457 ± 23	658 ± 36**	446 ± 29**	542 ± 26**
TAG (*μ*mol/L)	1196 ± 66	864 ± 51**	1018 ± 53**, ‡	994 ± 52
Total cholesterol (mmol/L)	6.0 ± 0.1	5.2 ± 0.1**	5.7 ± 0.1**, ‡	5.9 ± 0.1*
Adipocyte size (*μ*m)	68.5 ± 0.7	62.0 ± 0.7**	65.6 ± 0.7**, ‡	67.4 ± 0.7**
HOMA‐IR	3.75 ± 0.21	2.60 ± 0.16**	2.95 ± 0.18*, ‡	2.78 ± 0.25*
ACE activity (U/L)	46.5 ± 2.6	41.5 ± 2.4**	44.2 ± 3.4**	46.9 ± 2.7*
IL‐6 (pg/mL)	0.73 ± 0.05	0.75 ± 0.05	0.73 ± 0.05	0.64 ± 0.04
RBP4 (*μ*g/mL)	27.0 ± 1.1	23.7 ± 1.1**	26.2 ± 1.0**	27.4 ± 1.0*
Apelin (ng/mL)	0.63 ± 0.04	0.59 ± 0.04	0.65 ± 0.05	0.54 ± 0.04**
Leptin (ng/mL)	29.4 ± 2.7	13.2 ± 1.5**	17.7 ± 1.9**, ‡	24.6 ± 2.6**
Adiponectin (*μ*g/mL)	9.0 ± 0.7	9.4 ± 0.7*	10.0 ± 0.7*, ‡	9.9 ± 0.7
Vaspin (ng/mL)	0.34 ± 0.07	0.28 ± 0.05	0.38 ± 0.05**, ‡	0.36 ± 0.06
Nesfatin‐1 (ng/mL)	2.52 ± 0.71	2.64 ± 0.80	2.65 ± 0.78	2.68 ± 0.79

Values are mean ± SEM. *P <* *0.05, **P <* *0.01 change between this time point and previous time point. ^†^ *P *<* *0.05, ^‡^ *P *<* *0.01 change between the end of WS and study start (DI period). WL, weight loss; WS, weight stable; LCD, low‐calorie diet; VLCD, very‐low‐calorie diet; HOMA‐IR, homeostatic model assessment insulin resistance; ACE, angiotensin converting enzyme; RBP4, retinol‐binding protein 4; IL‐6, interleukin 6.

**Figure 2:**
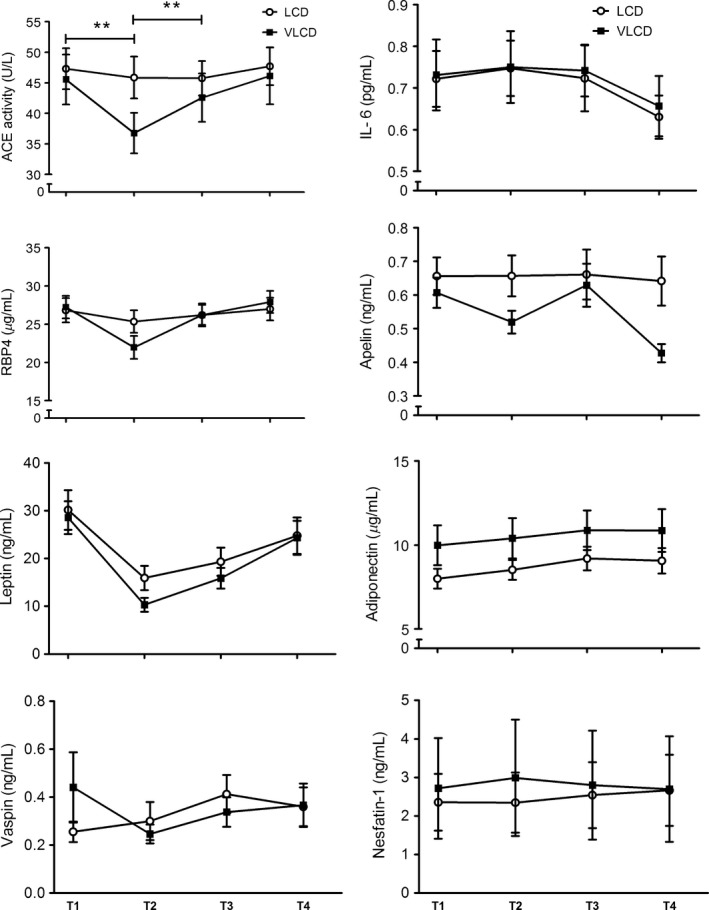
Concentrations of several adipokines and other biomarkers at T1, T2, T3, and T4 in the low‐calorie diet (LCD) group and very‐low‐calorie diet (VLCD) group. Significant diet × time interactions were observed for angiotensin‐converting enzyme (ACE) activity and RBP4. Post hoc analysis: **P <* *0.01, significantly different between dietary groups (Bonferroni corrected). Values are mean ± SEM.

The concentrations of ACE activity, IL‐6, RBP4, apelin, leptin, adiponectin, vaspin and nesfatin‐1 at T1, T2, T3, and T4 in both dietary groups are shown in Figure 2. Leptin concentrations decreased in the whole group and to a similar extent between dietary groups (Table 2 and Fig. 2). Significant diet × time interactions were observed for the variables: absolute fat‐free mass, glucose, 2‐h glucose, FFA, total cholesterol, ACE activity and RBP4 (Table 1 and Fig. 2). Post hoc analysis revealed that, with the exception of 2‐h glucose, all diet × time effects occurred during the WL and/or WS period (Table 1 and Fig. 2). The changes in 2‐h glucose were significantly different between dietary groups during the DI period (LCD: −0.99 ± 0.24 mmol/L; VLCD: 0.17 ± 0.27 mmol/L, *P *=* *0.014) and during follow‐up (LCD: 0.81 ± 0.38 mmol/L; VLCD: ‐1.45 ± 0.41 mmol/L, *P *=* *0.001) (Table 1). Although we observed a significant diet × time interaction for RBP4, changes in RBP4 concentrations were nonsignificant after Bonferroni correction over any time period. ACE activity decreased more strongly during WL and subsequently increased more strongly during WS in the VLCD group compared to the LCD group (WL period: −8.79 ± 1.29 U/L vs. −1.44 ± 1.70 U/L, respectively, *P *=* *0.008; WS period: 5.80 ± 1.13 U/L vs. −0.11 ± 1.22 U/L, respectively, *P = *0.005) (Fig. 2).

### Predictors of weight regain

**Figure 3:**
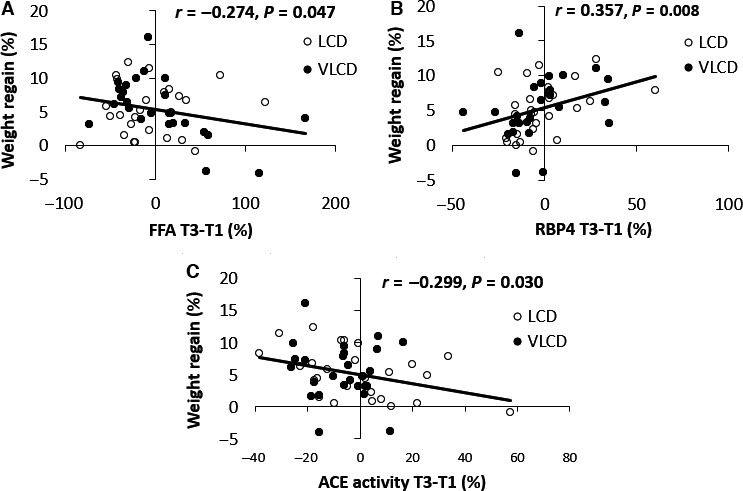
Correlations between weight regain (%) and percentage change in FFA (A) and RBP4 (B) concentrations and angiotensin‐converting enzyme (ACE) activity (C) during the DI period (T3–T1) in the low‐calorie diet (LCD) group and very‐low‐calorie diet (VLCD) group. The trendline, correlation (*r*) and *P*‐values are shown for the whole group.

To predict weight regain, changes in concentrations of biomarkers during the WL period (T2–T1) and DI period (T3–T1) were used. During the WL period, only changes in RBP4 were correlated with weight regain (Spearman's rho: 0.319, *P *=* *0.019). During the DI period changes in FFA, ACE activity and RBP4 were significantly correlated with weight regain (Fig. 3 and Table 3). This analysis was also performed for the LCD group and VLCD group separately, but showed similar results as analyzing the groups together (data not shown). Increases in ACE activity and FFA concentrations and decreases in RBP4 concentrations during the DI period were associated with less weight regain during follow‐up (*r* = −0.299, *P *=* *0.030; *r* = −0.274, *P *=* *0.047; *r* = 0.357, *P *=* *0.008, respectively) (Fig. 3). The correlation of FFA changes with weight regain was largely explained by participants from the VLCD group (Table 3). In contrast, changes in RBP4 and ACE activity during DI were better predictors of weight regain in the LCD group compared to the VLCD group (Table 3). Furthermore, changes in RBP4 and FFA during DI appeared better predictors of weight regain in men compared to women. In the whole group, weight loss during the DI period was not correlated with weight regain (*r* = 0.020, *P *=* *0.884) and was therefore not used as a predictor of weight regain. Concentrations at T1, T2, T3 or T4 of FFA, TAG, total cholesterol, insulin, glucose, HOMA‐IR, ACE activity, IL‐6, RBP4, apelin, leptin, adiponectin, vaspin and nesfatin‐1, and adipocyte size were not correlated with weight regain (data not shown). Changes in concentrations of adipokines and other biomarkers during the DI period are more likely to reflect chronic changes in homeostasis than those over the WL period, because they represent weight loss‐induced changes without the presence of a negative energy balance. Therefore, we tested the predictive value of concentration changes over the DI period for weight regain. Using a backward elimination regression model (variables entered: age, sex, diet, BMI at T1 and DI‐induced changes in ACE activity, RBP4 and FFA) we showed that changes in ACE activity, FFA and RBP4 during DI (independent) were all significantly correlated with weight regain (dependent) (Table 4). This model explained 28% (*r* = 0.532, *P = *0.001) of the variation in weight regain. The variables age, sex, diet and BMI at T1 did not significantly improve the model and were excluded (*P *>* *0.1). RBP4 changes during the WL period were not entered into the model since this would overlap with the RBP4 changes during the DI period, which was also a stronger predictor. Finally, the VLCD group and LCD group were analyzed separately.

**Table 3:** Correlations between % changes in ACE activity, FFA and RBP4 concentrations (T3–T1) with weight regain (%) during follow‐up (T4–T3)

		Whole group (*n* = 56)	LCD (*n* = 29)	VLCD (*n* = 27)	Women (*n* = 30)	Men (*n* = 26)
Correlation weight regain changes T4–T3 (%) with:						
FFA change T3–T1 (%)	*r* *P*‐value	−0.274 0.047^1^	0.044 0.826	−0.565 0.003^1^	−0.184 0.359	−0.313 0.120
RBP4 change T3–T1 (%)	*r* *P*‐value	0.357 0.008^1^	0.421 0.026^1^	0.302 0.134	0.396 0.037^1^	0.376 0.058
ACE activity change T3–T1 (%)	*r* *P*‐value	−0.299 0.030^1^	−0.444 0.020^1^	−0.119 0.564	−0.184 0.359	−0.492 0.013^1^

^1^Indicates significant correlation at *P *<* *0.05 level. WL, weight loss; WS, weight stable; LCD, low‐calorie diet; VLCD, very‐low‐calorie diet; ACE, angiotensin converting enzyme; RBP4, retinol‐binding protein 4.

**Table 4:** Weight regain is predicted by a model with RBP4, ACE activity, and FFA change during DI using backward elimination in multiple linear regression

	*B*	SE *B*	*β*	*P*‐value
RBP4 change T3–T1 (%)	0.068	0.024	0.339	0.007
ACE activity change T3–T1 (%)	−0.067	0.028	−0.292	0.020
FFA change T3–T1 (%)	−0.023	0.010	−0.273	0.029
Model *R* ^2^ = 0.283, *P *=* *0.001				

ACE, angiotensin converting enzyme; RBP4, retinol‐binding protein 4; SE, standard error.

## Discussion

In this study we selected adipokines and other biomarkers that were previously shown to be correlated with weight regain or had the potential to influence weight regain due to functions related to energy expenditure, food intake or body weight. Here, we show for the first time that dietary weight loss‐induced changes in RBP4 and FFA concentrations independently predict weight regain over a 9‐month follow‐up period in people with overweight and obesity. Furthermore, and in contrast to previous observations, changes in ACE activity were negatively correlated with weight regain. These three predictors independently contribute to weight regain prediction, indicating they largely act on separate underlying mechanisms.

RBP4 is a transport protein for retinol (Vitamin A) with the highest expression in liver and the second highest expression in AT (Tsutsumi et al. 1992). We have recently shown in the Diogenes study that higher baseline RBP4 correlated with increased regain of lost weight in people with obesity (Wang et al. 2013). Moreover, the minor allele of a RBP4 regulatory SNP has been found to enhance adipocyte RBP4 expression and to be correlated with a higher BMI in an Asian population (Munkhtulga et al. 2010). Here, we report the novel finding that dietary weight loss‐induced changes in RBP4 are positively correlated with weight regain during follow‐up. The mechanism linking RBP4 with body weight is largely unclear, although it has been speculated that RBP4 stimulates adipocyte proliferation, which could increase weight regain (Munkhtulga et al. 2010; Wang et al. 2013). However, more mechanistic studies are needed to confirm this hypothesis.

ACE has been implicated in obesity because ACE polymorphisms were associated with body weight and obesity incidence (Lagou et al. 2007; Bouwman et al. 2014). Furthermore, higher levels of the product of ACE, angiotensin II (Ang II), stimulates adipocyte differentiation (Frigolet et al. 2013) and could thereby affect adipose tissue mass. As mentioned earlier, we previously observed in the Diogenes study that a greater reduction in ACE concentration during weight loss was associated with less body weight regain during the maintenance period in men and women (Wang et al. 2011, 2012). Animal studies show an effect of ACE inhibitors on body weight regulation by decreasing body water content (Toritsuka et al. 1999) or through a reduction in energy intake (de Kloet et al. 2009; Velkoska et al. 2010). Surprisingly, in the current study a greater reduction in ACE activity during the DI was correlated with increased weight regain. Some reports are in line with this outcome, for example, chronic intracerebroventricular administration of Ang II resulted in weight loss in young rats (Porter et al. 2003). Similarly, transgenic mice with increased brain‐specific renin angiotensin system activity were lean due to increased energy expenditure compared to wild‐type mice (Grobe et al. 2010). An important difference between the results in the Diogenes study and this study is that the correlation in this study was observed when a 4‐week weight stable period was included, in which the ACE activity returned toward levels at study start. Furthermore, in this study only three participants continued to lose weight during follow‐up, whereas from the Diogenes study subjects were selected for analysis that were at the extremes of weight loss maintenance or regain. Thus, 48 out of 123 selected participants continued to lose weight during the follow‐up period. The exact reason for the contradictory findings in our ACE‐related studies remains elusive, but stresses the need for more causal clinical and mechanistic studies to depict the precise role of ACE in body weight regulation.

In this study increases in FFA concentrations during DI were correlated with less subsequent weight regain. The potential role of FFAs in energy homeostasis, food intake and insulin resistance has been extensively studied and involves different pathways. Central administration of oleic acid reduces food intake in rats, probably by signaling nutrient abundance within the central nervous system (Obici et al. 2002; Morgan et al. 2004). More recently it was shown that FFAs of any length can directly function as signaling molecules through G‐protein coupled receptors. These receptors are distributed throughout a wide variety of tissues with functions in energy balance, regulation of appetite and insulin resistance (Ichimura et al. 2009; Ulven 2012). GPR120 is a long‐chain FFA receptor expressed in various tissues including intestine and AT that has a crucial role in adipogenesis, appetite regulation and obesity (Gotoh et al. 2007; Ichimura et al. 2012). Additionally, Ichimura et al. demonstrated that a deleterious mutation (p.R270H) inhibits GPR120 signaling and increases the risk of obesity (Ichimura et al. 2012). The short‐chain free fatty acid receptors FFAR2 and FFAR3 are important for lipolysis, adipogenesis, PYY release from the gut and leptin production (Hong et al. 2005; Ichimura et al. 2009), and could thereby influence energy metabolism and body weight. Interestingly, the correlation between FFA and weight regain was much stronger in the VLCD group compared to the LCD group, yet on average no differences in FFA changes during DI were observed between groups. Importantly, since FFA concentrations are closely related to AT metabolism, the correlation of weight loss‐induced changes in FFA and weight regain could also be associated with changes in AT metabolism, which might contribute to variations in weight regain, rather than the effects of FFA on FFA receptors.

Interestingly, the variables that were associated with weight regain, RBP4, ACE activity and FFA, were all independent predictors of weight regain. When we consider the largely distinct mechanisms to influence body weight of these three predictors as shown above, it is perhaps not surprising they are independent predictors of weight regain. Together this panel accounts for 28% of weight regain variation. Other studies that have predicted weight regain using single variables: dietary restraint (22%), resting metabolic rate at baseline (14%), amplitude of body weight (5%) and diet‐induced changes in leptin concentrations (13%) have shown similar or less predictive power (Vogels et al. 2005; Erez et al. 2011). It would be interesting to investigate whether a combination of these variables can increase weight regain prediction. However, the practical implications of our results alone are limited since most of the weight regain variability is still unaccounted for.

Previous studies in humans have identified leptin and IL‐6 as predictors of weight regain (Goyenechea et al. 2006; Crujeiras et al. 2014). Conversely, and in line with our results, a recent review concluded that there was little evidence that dietary weight loss‐induced changes in leptin or pre‐ or postweight loss leptin could predict weight regain (Strohacker et al. 2014). Gene polymorphisms in IL‐6 were related with weight regain after weight loss (Goyenechea et al. 2006) and recently it was shown that high plasma IL‐6 concentrations were associated with short‐term (6 week) weight regain in humans (Kong et al. 2013). Such an association was not observed in the current study and given the crucial function of IL‐6 in the inflammatory response, the effects of IL‐6 on weight regain might be secondary to inflammation. Furthermore, we investigated the role of vaspin, apelin, and nesfatin‐1 in weight regain since they were shown to play a role in mitochondrial biogenesis (Attane et al. 2012), energy expenditure (Higuchi et al. 2007; Stengel et al. 2009) and/or food intake (Brunetti et al. 2011; Kloting et al. 2011) in animal studies, and could thereby influence weight regain. In most of these studies the animals were infused with these substances, leading to drastic phenotypical changes. Here, we studied the effects of more subtle dietary weight loss‐induced changes in these concentrations and this could explain why we did not observe an association with weight regain.

In this study a rather heterogeneous population was used regarding sex, menopausal status, age and other variables, which could have influenced our results. Furthermore, our conclusions are based on associations between variables, which have limited predictive power. Future studies can be designed to investigate the potential causal relationship between diet‐induced changes in RBP4, FFA, and ACE activity, and weight regain.

In conclusion, we demonstrate that weight loss‐induced increases in RBP4 and decreases in FFA concentrations predict unfavorable long‐term weight changes in people with overweight and obesity. The reason why the correlation between ACE activity and weight regain was in the opposite direction compared to our previous study is unclear, although weight regain patterns during follow‐up were different between studies. Together, this panel of ACE activity, RBP4 and FFA concentrations were independently correlated with weight regain, indicating they act on separate underlying mechanisms and implies they could be further investigated as potential targets for weight management.

## Conflict of Interest

The authors declare no conflict of interest.
